# COVID-19 impact and response by Canadian pain clinics: A national survey of adult pain clinics

**DOI:** 10.1080/24740527.2020.1783218

**Published:** 2020-09-10

**Authors:** Mary E. Lynch, Owen D. Williamson, Jillian C. Banfield

**Affiliations:** a Anesthesia, Pain Management and Perioperative Medicine, Pain Management Unit, Dalhousie University, Halifax, Nova Scotia, Canada; b Department of Anesthesia, McMaster University, Hamilton, Ontario, Canada; c Anesthesia, Pain Management and Perioperative Medicine, Dalhousie University, Halifax, Nova Scotia, Canada

**Keywords:** COVID-19, pain clinics, distance treatments, e-health, telehealth

## Abstract

**Background**: As the result of public health authority responses to the COVID-19 pandemic, pain clinics have had to cease providing in-person appointments to reduce contact between patients and staff. Over the past decade, Canadians living with chronic pain have faced long waiting times for care within multidisciplinary pain clinics. We are concerned that ceasing in-person pain services exacerbates the daily hardships already faced by Canadians living with chronic pain.

**Aims**: The aim of this study was to evaluate the impact of the COVID-19 pandemic on Canadian pain clinics, their responses, and changes to clinic practices that might be maintained when the pandemic is over.

**Methods**: A survey of Canadian adult multidisciplinary pain clinics was conducted to determine impacts on medical and allied health care services and the strategies used to deliver care to patients during the COVID-19 pandemic.

**Results**: Responses received from 17 adult pain clinics across Canada showed that adult multidisciplinary pain clinics had to cease or significantly reduce in-person patient contacts during the COVID-19 pandemic and responded by offering telehealth options. Despite their efforts, patients are waiting longer and have lost access to usual care. Increased levels of pain, stress, and medication use, particularly opioids and cannabinoids, were reported.

**Conclusions**: Access to adaptable and innovative technologies, such as telehealth, can assist in the care of the one in five Canadians living with chronic pain during times of crises and must be included as a vital component of a comprehensive Canadian pain strategy.

## RÉSUMÉ

**Contexte:** Suite aux mesures prises par les autorités de santé publique en réponse à la pandémie de COVID-19, les cliniques antidouleur ont dû cesser de proposer des rendez-vous en personne afin de réduire les contacts entre les patients et le personnel. Au cours de la dernière décennie, les Canadiens vivant avec la douleur chronique ont dû faire face à de longs délais d’attente pour obtenir des soins dans les cliniques antidouleur multidisciplinaires. Nous sommes préoccupés par le fait que l’arrêt des services antidouleur en personne exacerbe les difficultés quotidiennes auxquelles sont déjà confrontés les Canadiens qui vivent avec la douleur chronique.

**Objectifs:** Cette étude visait à évaluer les repercussions de la pandémie de COVID-19 sur les cliniques antidouleur au Canada, leurs réactions et les changements dans leurs pratiques qui pourraient être maintenus en raison de la pandémie.

**Méthodes:** Une enquête a été menée auprès des cliniques antidouleur multidisciplinaires pour adultes au Canada afin de determiner les répercussions sur les services médicaux et les services paramédicaux, ainsi que les strategies utilisées pout fournir des soins de santé aux patients pendant la pandémie de COVID-19.

**Résultats:** Les réponses reçues de 17 cliniques antidouleur pour adultes au Canada ont montré que les cliniques antidouleur multidisciplinaires pour adultes avaient dû cesser ou réduire considérablement les contacts en personne avec les patients pendant la COVID-19 et qu’elles ont réagi en proposant des options de télésanté. Malgré leurs efforts, les patients attendent plus longtemps et n’ont plus accès aux soins habituels. L’augmentation des niveaux de douleur, de stress et de médication, en particulier les opioïdes et les cannabioïdes, a été signalée.

**Conclusion:** L’accès à des technologies adaptables et innovantes, telles que la télésanté, peut contribuer aux soins des 20 % de Canadiens vivant avecla douleur chronique en période de crise et doit constituer un élément essentiel d’une stratégie canadienne globale de lutte contre la douleur.

## Introduction

Across Canada, in order to comply with government directives for physical distancing, multidisciplinary pain clinics have ceased providing in-person contact between patients and staff in order to help prevent the spread of the novel coronavirus SARS-CoV-2. Over the past decade, people living with pain have already faced long waiting times for the assessment and management of their pain in these clinics.^1^ In March 2019, the federal government established the Canadian Pain Task Force. In June 2019, the Canadian Pain Task Force’s initial report confirmed that Canadians have inadequate access to pain services and that waiting times for available services are long, resulting in devastating impacts on individuals living with pain, their families, communities, and the Canadian economy.^2^ The COVID-19 pandemic has led to major changes in delivery of pain care internationally. A recent review discussed the international impact of COVID-19 on pain treatment centers, the adverse consequences of not treating chronic pain, and strategies for the rapid introduction of remotely supported pain services.^3^

Based on clinical experience, we predicted that ceasing in-person pain services would exacerbate the daily hardships faced by the Canadians living with chronic pain. We aimed to study the impact of the COVID-19 pandemic on Canadian pain clinics, how they responded, and what changes may be maintained when the pandemic is over. This study involved a survey designed to explore the impact on the ability of Canadian adult multidisciplinary pain clinics to provide care and the strategies they are using to deliver care to patients during the COVID-19 pandemic.

## Materials and Methods

**Figure 1.**
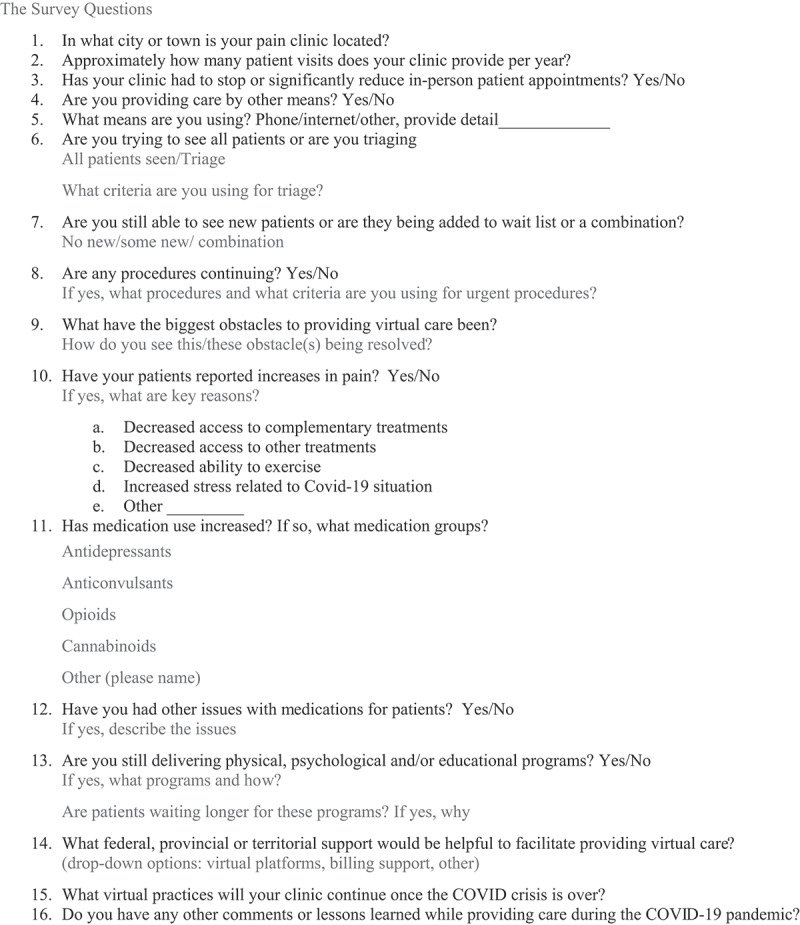
The survey questions

A survey was developed to explore the initial impact and experiences of Canadian pain clinics in the context of COVID-19 pandemic (Figure 1). The survey included questions about the location and activities of the clinics, the impact on services that were previously offered, impacts on patients, and whether new initiatives would continue after the pandemic. The survey was approved by the Nova Scotia Health Authority Research Ethics Board (#1025673) and informed consent was obtained from those completing the survey. The survey was distributed by e-mail to members of the Academic Pain Directors of Canada (APDOC), an association of the medical directors of university and regional health authority multi-/interdisciplinary pain clinics across Canada. A secure web application (REDCap) was used for building, collecting, and managing the survey data. Participants were asked to complete the survey within 2 weeks, and reminder e-mails were sent out after 1 week. Data were de-identified and aggregated.

## Results

Surveys were sent to 29 members of APDOC representing 20 adult pain clinics and the medical directors of three additional university-affiliated multidisciplinary pain clinics who had been invited to become members of APDOC prior to completing the survey. One accepted the invitation (Kelowna) and completed the survey. We requested that only representatives of adult clinics respond to this survey and that an effort be made for each clinic to complete one survey. Data were collected between April 23 and May 5, 2020. There were 18 responses. One response was from a pediatric clinic and was excluded, so the final response rate was 17/23 (73.9%). Every question was answered by all responders. What follows is the descriptive data from the 17 responses.

Responses were received from across the country, with representation from Vancouver, Kelowna, Calgary, Edmonton, Winnipeg, Hamilton, Greater Toronto, London, Kingston, Montreal, Quebec City, and Halifax. The number of first visits and follow-up visits to clinics ranged from 400 to 23,000 per year, with six clinics reporting 10,000 or more visits, five clinics reporting 5000 to 9999, and six reporting less than 5000 visits per year. All clinics indicated that they had to cease or significantly reduce in-person clinic appointments, and all clinics reported providing care by other means. All were providing phone contact and 11 were providing additional options, including Zoom for Healthcare or nonspecified video options. Fifteen clinics (88%) were able to do assessments on some new patients; however, 13 clinics (76%) were also adding new referrals to a wait list. Eleven clinics (65%) reported that patients were waiting longer for care. Nine clinics (53%) were still trying to see all patients and eight clinics (47%) were using some form of triage (see Table 1). All but one clinic provided physiotherapeutic, psychological, or educational services using phone or video technology. Most services were provided individually, but one site was offering group mindfulness and art therapy, and another two sites were about to commence group programs remotely. Six (35%) clinics were providing no procedures and 11 (65%) were providing limited access to procedures (see Table 2). Thirteen clinics (76%) reported obstacles to providing virtual care, including lack of sufficient equipment, lack of administrative support, and delays in accessing authorized telehealth platforms (Table 3). Most reported that with time, experience, and access to staff, some of whom had been redeployed away from pain clinics, the issues were being gradually resolved.

**Table 1.** Responses to question “Are you still trying to see all patients or are you triaging? What criteria are you using for triage?”

Still seeing all patients	9 clinics
Triaging	8 clinics
Triage responses	Clinic was totally closed for 4 weeks … no phone no nothing. We are starting back with patients who do not need ‘only’ an infiltration, and we will also call those patients if visits in person cannot start back
	Deferred patient list contacted and asked if they can wait
	Not determined yet
	Urgent cases; that is, life or limb. If patient will come to harm without treatment within 6 weeks (e.g., intrathecal pump fills, infections, malfunctioning neurostimulators, or acute CRPS less than 3 months)
	Patients offered the opportunity to discuss pain management treatment with clinicians. Emphasis placed on medication and self-management strategies
	Absence of COVID-19 symptoms. Need for urgent evaluation (e.g., the patient will come to the emergency if his condition is not addressed). Need to prescribe opioid
	Patients with cancer are prioritized for assessment and treatment; patients who are essential workers are also prioritized for treatments (such as injection therapies)
	We prioritize cancer patients, acute radicular pain, acute CRPS, and patients in severe pain. We see patients that will otherwise go to ER. We postpone treatments on elderly with multiple comorbid conditions

CRPS = chronic regional pain syndrome; ER = emergency room.

**Table 2.** Answers to question “Are any procedures continuing? If yes, what procedures and what criteria are you using for urgent procedures?”

No procedures	6 clinics
Yes procedures	11 clinics
Criteria for urgent procedures	Cancer for both inpatients and outpatients
	Procedures for hospitalized patients (e.g., ketamine infusion, lidocaine infusion, some spinal injections)
	Urgent only; for example, patients who would otherwise be at risk of accessing acute care services to manage pain flares
	Intrathecal drug delivery systems only
	Intrathecal pump fills, malfunctioning stimulators, and blocks for acute CRPS
	Intrathecal pump refills. All other treatments at this point are deferred
	Very few procedures for specific indications (severe pain despite optimized medical treatment). No corticosteroids
	Cancer-related interventional procedures are being performed, as are injection therapies for patients with headache, as well as trigger point injections for essential workers
	Nonsteroid injecting procedures including neurotomy, pulsed radiofrequency, local anesthetic injections
	Only patients in severe pain: acute radicular pain (epidurals or nerve root blocks) or neuropathic pain (post herpetic neuralgia), acute facet joint pain for medial branch blocks and rhizotomies
	College of Physicians and Surgeons Alberta guidelines

CRPS = chronic regional pain syndrome.

**Table 3.** Answers to question “What have the biggest obstacles to providing virtual care been? How do you see these obstacles being resolved?”

Biggest obstacles	How resolved
(1) Insufficient equipment in the hospital; (2) unclear billing policies; (3) elder, poorer, and lower educated patients with harder access to virtual means; (4) administrative coordination is challenging; (5) insufficient practice-based guidelines/recommendations	Except for (3), all are being resolved slowly
Getting the “OK” from our chief to start back (confusing information for hospital authorities). Getting lists of patients while at home. Getting access to the electronic files of the patients (took 2 weeks!)	Finally got the OK (!) Found a way by password protected pdf to exchange information, but it takes a lot longer to do simple tasks
None	
New patients	Not resolved
Hospital support	Time
Mainly provider comfort with the technology. To be honest, most patients are more comfortable with it than we are	Experience/practice
None	
Slow process for technology selection, program development, and ensuring availability of staff (some have been redeployed to COVID-related activities. Management attention is focused on COVID related responsibilities, clinic is low priority.)	We have identified available staff now and tech platform(s). Program development underway, 6 weeks into the process
New patients cannot be examined physically	I opened up the clinic to see new patients who want to come in last 2 weeks
None really, we have good technology. Some patients aren’t that facile with smartphones, or don’t have them, so we use landline telephone calls for that	
Physical assessment, patient access, and ability to use IT	Offer telephone visit. Will see patient in person at later date for physical assessment
New platform. Some patients do not have access to Internet	The more we practice this, I expect the easier it will become
Adequate access to the authorized platforms	Time
Actually, the provision of virtual care has been facilitated in a number of ways: (1) The provincial government has created billing codes for such services; (2) patients have been receptive to being contacted by phone or Zoom and have found this to be more practical than coming in to the hospital, finding parking, etc.; (3) the clinic admin staff have facilitated setting up Zoom accounts for the physicians	
Approving secure formats to conduct virtual visits	Administration working on these issues to ensure secure formats
No real obstacle	
Cannot examine or do other procedures, no rehab, social isolation	Focus on other strategies; more frequent contact

Thirteen clinics (76%) indicated that their patients reported increased pain levels. When asked about the key reasons for the increase in pain, 11 clinics (65%) reported that patients had decreased access to complementary treatments and 12 clinics (71%) reported decreased access to other treatments. Twelve clinics (71%) reported that pain levels might be increased due to increased stress related to COVID-19 issues; three clinics (18%) reported stress related to other issues, including social isolation, decreased activity, finances, layoffs, grief, and access to treatment; and six clinics (35%) reported patient decreased ability to exercise.

Nine clinics (53%) reported that patient medication use had increased, with eight clinics (47%) reporting an increase in opioid and cannabinoid use, five clinics (29%) reporting an increase in anticonvulsant use, and four clinics (24%) reporting an increase in antidepressant use. Other medications noted to be increased included benzodiazepines (one clinic) and nonsteroidal anti-inflammatory drugs (two clinics). Four clinics reported other medication-related issues, including shortages, difficulty accessing medication, inability to do urine testing, and problems refilling opioid prescriptions. One clinic mentioned that pharmacists’ abilities to take phone orders on opioid prescriptions made it easier.

When asked what federal, provincial, or territorial support would be helpful to facilitate virtual care, 76% endorsed virtual platforms, 47% endorsed billing support, and 29% made additional suggestions (Table 4). The majority reported that they thought that virtual health care would continue even after the COVID-19 crisis is over, with several mentioning that appropriate billing support would facilitate this (Table 5). Under other takeaways, clinics reported that patients appreciated the option of phone or other virtual contact (Table 5).

**Table 4.** Answer to question “What federal, provincial, or territorial support would be helpful to facilitate providing virtual care?”

Virtual platforms	3 clinics
Billing support	8 clinics
Other forms of support	Allowing secure access to patient records from personal devices, as happens in other provinces. Providing limited access only through health authority–owned computers is very restrictive
	Helping patients with higher needs accessing virtual care
	To be able to bill for phone visits or consultations. To have access to “anonymous” e-mails for the clinic to send information that is not from our perennial mail
	Allowing me to resume my operations in full. Medical consults is a small part only of what we do
	We have just been approved for new and follow-up tariffs, which are the same as for in-patient clinic visits

**Table 5.** Answers to question “What practices will your clinic continue once the COVID-19 crisis is over? Do you have any other comments or lessons learned while providing care during the COVID-19 pandemic?”

After COVID-19	Lessons learned
Telemedicine, no doubt	
Phone follow-up if possible to bill (was not possible before; we have a basic amount and phone follow-ups are supposed to be included, but it takes as long as a regular visit in person!)	Patients appreciate support on the phone. We should continue using this modality
More virtual visits for follow-ups	
Phone follow-up	
Virtual clinic for patients who are far away	
Likely all of our new virtual options will continue for patients	Many patients prefer the virtual encounters and are not interested in going back to driving/parking
Not clear yet	No
It will partly depend upon billing practice changes at the provincial level	
We are preparing some additional online services for the future	Yes, it has made us more creative to add online services while we retain all that we do in the premises
The appetite for virtual follow-ups is huge among patients. Provided the billing codes remain on par, I can see that becoming the default method for simple follow-ups	
Some video follow ups if supported by government	
Virtual follow-up of patients with stable conditions, who live far from the hospital. Control visits after a procedure, if billing permits	
It is likely that follow-up visits will continue; it is possible that our clinic’s group medical visits may be delivered by virtually	We have seen that patients have demonstrated tremendous understanding about the challenging situation of not being able to see them in clinic, perform scheduled interventional procedures, etc. They have appreciated being contacted by telephone. Other patients have not been coping well and we have seen some patients contacting our nurses and physicians for increased psychological support
If billing tariffs remain we will continue to provide telephone follow-up and possibly initial new patient assessment by telephone	Patients have been very kind and supportive to clinic staff. Patients are very keen to continue with telephone follow-ups. Staff have been very supportive to each other. Great to see people come together and work together to the benefit of all
Initial consults for rural patients. Group counseling for our health region, which is the size of Texas with only one pain center	
Continue Zoom; continue online	Using it as an opportunity to promote other self-management strategies

## Discussion

This study found that adult multidisciplinary pain clinics across Canada have had to cease or significantly reduce in-person patient appointments during the COVID-19 pandemic, and every clinic responded by offering telehealth options including phone and Internet-based video appointments (most commonly Zoom for Healthcare). Despite these efforts, patients do not have access to usual care and are waiting longer for care at pain clinics. Only the most urgent procedures have continued, leaving many patients without access to other diagnostic or therapeutic interventional procedures. The majority have had no access to complementary therapies such as acupuncture, osteopathy, chiropractic treatment, or hands-on physiotherapy. Patients’ pain and stress levels were reported to be higher. Medication use has escalated, with most frequent increases being reported for opioids and cannabinoids but also including antidepressants and anticonvulsants.

Previous work has identified that people living with chronic pain conditions have very poor quality of life, with 50% experiencing severe or extremely severe levels of depression and 35% experiencing suicidal ideation.^4^ There is a significant deterioration in health-related quality of life and psychological well-being while waiting for care, including increasing pain and depression.^5^ It is clear that the public health response to the COVID-19 pandemic requiring pain clinics to cease in-person contact has caused significant harm to people living with chronic pain. The negative impact on their care persists despite creative efforts by clinic staff across the country to provide telehealth options. The current pandemic has accelerated the support and uptake of telehealth technologies, which is an improvement in delivery of care that should continue. These technologies will be useful when the COVID-19 pandemic is over. Telehealth is often more convenient for patients and provides additional, more cost-effective options for follow-up care.

A key limitation to this study is that it did not collect data directly from patients. This survey was answered from the perspective of the clinic directors or their designates. It will be important in a future study to obtain information directly from people living with pain conditions. We identified participants through APDOC membership, in order to survey a convenient sample over a time frame that enabled us to rapidly communicate possible solutions to the challenges that multidisciplinary pain clinics face in this time of crisis. Because most of the clinics involved in this study are large university-affiliated facilities, the challenges faced and solutions offered may not represent those of all multidisciplinary pain clinics across Canada.

Public health authorities must consider possible harms to people living with chronic pain when they consider their responses to the current and future pandemics. Further limiting access to care will exacerbate the impact of chronic pain on individuals, their families, communities, and the Canadian economy.^2^ Only time and further study will tell the full story of the costs of the public health response to COVID-19 on people with chronic pain. Though it is vital to ensure that the health system can deal with the acute impacts of COVID-19, it is equally important that in planning for future crises we address the need to continue the treatment of chronic conditions and disease prevention programs, such as vaccination and cancer screening, in order to decrease ongoing morbidity and excess mortality due to these conditions. We must build upon the innovative approaches to improving the care of people living with pain that the multidisciplinary pain clinics have started or accelerated during COVID-19. We recommend that the Canadian Pain Task Force advise Health Canada that a coordinated approach to the management of chronic pain in times of crisis be included as a vital component of a comprehensive Canadian pain strategy.
